# Laparoscopic assisted dismembered pyeloplasty versus open pyeloplasty in UPJO with poorly function kidney in pediatrics

**DOI:** 10.1007/s11255-023-03799-4

**Published:** 2023-09-26

**Authors:** Mohammad Daboos, Rachida Lamiri, Bochra Aziza, Mohamed Marzougui, Nahla Kechiche, Lassaad Sahnoun, Mohamed Abdelaziz, Mohamed Abdelmaboud, Ahmed Azab, Mahmoud Shaban

**Affiliations:** 1 Department of Pediatric Surgery, Al-Azhar University Hospitals, Cairo, Egypt; 2 Department of Pediatric Surgery, University of Monastir, Monastir, Tunisia

**Keywords:** Pediatric UPJO, Laparoscopic assisted pyeloplasty, Open pyeloplasty, Poorly functioning kidney

## Abstract

**Background:**

The management of UPJO with poor function kidney, less than 10%, has been the subject of debate for more than a decade. Some authors have recommended nephrectomy, while others favor renal salvage (pyeloplasty). We report our experience with laparoscopic assisted pyeloplasty in pediatric patients with poorly functioning kidneys in comparison with an open approach.

**Materials and methods:**

A retrospective study was conducted to review 65 patients who were diagnosed with hydronephrosis and had impaired renal function due to UPJO. The study was conducted in the pediatric surgery departments of Al-Azhar University Hospital and Fattouma Bourguiba University Hospital of Monastir over a period of 20 years. Limited to pediatric patients with UPJO with ≥ Grade III hydronephrosis, antero-posterior pelvic diameter ≥ 20 mm, as well as a renal function equal to or less than 10%, was corrected by laparoscopic assisted or open pyeloplasty.

**Results:**

There were 40 cases in group A who underwent laparoscopic assisted pyeloplasty, and 25 cases in group B who underwent open pyeloplasty. There were no complications or difficulties during the operation. The mean operative time in group A was 90 ± 12 min, while in group B, it was 120 ± 11 min. The renal assessment parameters significantly improved in both groups. In group A, the mean split renal function was 7.9 ± 1.3% and increased to 22.2 ± 6.3%. In group B, the mean split renal function was 8.1 ± 1.1% and increased to 24.2 ± 5.1%. However, the differences between both groups in terms of pre-operative and post-operative renal functions were statistically insignificant.

**Conclusion:**

Laparoscopic assisted pyeloplasty is an effective treatment for patients with poorly functioning kidneys, especially those with less than 10% function. While this surgical procedure requires shorter operative times, it yields functional outcomes that are comparable to open approach.

## Introduction

Ureteropelvic junction obstruction (UPJO) is the most common type of obstructive malformative uropathy [1]. It has been reported that the condition affects approximately 1 in every 500 births [2]. The management of UPJO in children has undergone significant advancements in the past 2 decades, primarily due to the introduction of antenatal real-time ultrasound and dynamic scintigraphy [3].

Fetal ultrasound has facilitated the timely identification and treatment of renal deterioration, preventing its progression to an advanced stage. Dynamic renal scintigraphy has enabled the estimation of the impact on renal function and the extent of obstruction [4, 5].

Once the diagnosis of UPJO has been established, the assessment of kidney function should guide the overall management approach. This may involve therapeutic abstention and monitoring of the junctional anomaly, or conservative treatment options such as laparoscopic or open pyeloplasty, or radical treatment approaches such as nephrectomy [2, 6]. The management of kidneys exhibiting poor function, specifically less than 10%, has been the subject of debate for over a decade. Some authors have recommended nephrectomy, while others favor renal salvage (pyeloplasty) [7].

We reported our experience with laparoscopic assisted pyeloplasty in pediatric patients with poorly functioning kidneys. Our focus was on the evaluation of functional outcomes and the advantages of using a minimally invasive approach for repairing UPJO in comparison with an open approach.

## Materials and methods

A retrospective analysis was conducted on 65 patients who were diagnosed with hydronephrosis and had poor renal function due to UPJO. The study took place at the Pediatric Surgery Department of Al-Azhar University Hospitals and the Pediatric Surgery Department of Fattouma Bourguiba University Hospital of Monastir. The review covered the period from January 2003 to May 2023. We collected data from hospital records on the clinical presentation, investigations, operative procedures, and post-operative outcomes of patients who were diagnosed as UPJO with Grade III hydronephrosis, an antero-posterior pelvic diameter greater than 20 mm, renal function equal to or less than 10% and treated by laparoscopic assisted dismembered pyeloplasty or Open dismembered pyeloplasty. Patients with renal function greater than 10%, recurrent cases, cases with ureteral dilatation (VUR), Duplex kidney, and cases treated by other types of pyeloplasty, were not included in this study. Our institutional review board approval was obtained and numbered (IRB 8-01-2022-000048). All procedures were performed after the parents signed a written informed consent and adhered to the ethical principles outlined in the Declaration of Helsinki. The study has been registered at (ClinicalTrials.gov) and numbered as [NCT05953571]*.*

All procedures either laparoscopic assisted pyeloplasty or open pyeloplasty were performed by two surgical teams, and each team included 5 surgeons, who are also the authors of this article. They possess over 20 years of experience in managing this particular issue and other urological diseases in pediatric patients.

### Operative technique

#### Group A (laparoscopic assisted pyeloplasty)

**Fig. 1:**
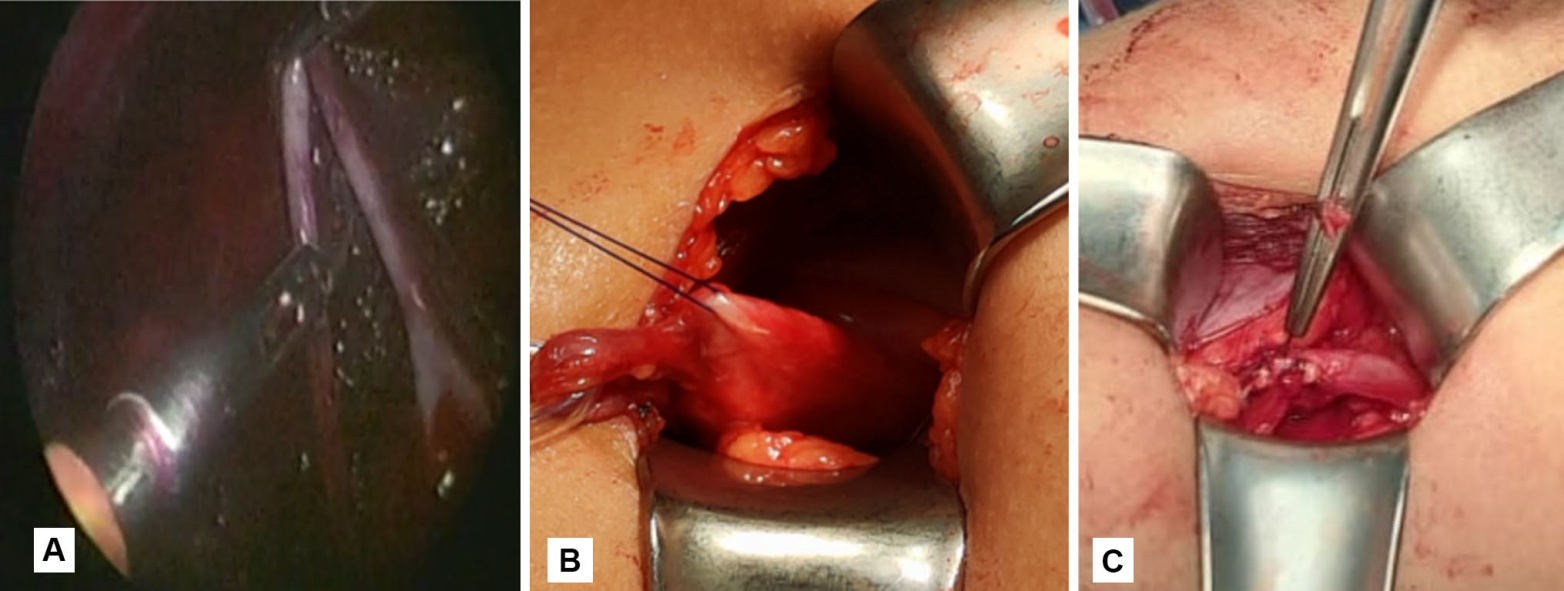
**A** Mobilization of the renal pelvis and ureter. **B** Exteriorization of the renal pelvis and UPJ. **C** New UPJ was dropped back into the peritoneal cavity

Surgery was performed under general anesthesia using endotracheal intubation, with lumbar padding to hyperextend the trunk obliquely. Three ports were used in all children: a 5-mm umbilical camera port and 5-mm working ports along the anterior axillary line in the subcostal and iliac regions. The kidney was exposed after medially reflecting the colon. After completing laparoscopic mobilization of the UPJ (Fig. 1A), a stitch was placed to secure the redundant pelvis to the abdominal wall, including the renal pelvis and the proximal ureter. The UPJ then easily exteriorized from the port site after a 2 cm lateral extension of the wound (Fig. 1B). With loupe magnification, a formal dismembered Anderson–Hynes repair was performed through the small incision using 5/0 absorbable suture. Antegrade stenting was done after completing the posterior layer of the anastomosis, the new UPJ was dropped back into the peritoneal cavity (Fig. 1C), a tube drain was left in the operative bed and the wound was closed.

#### Group B (open pyeloplasty)

**Fig. 2:**
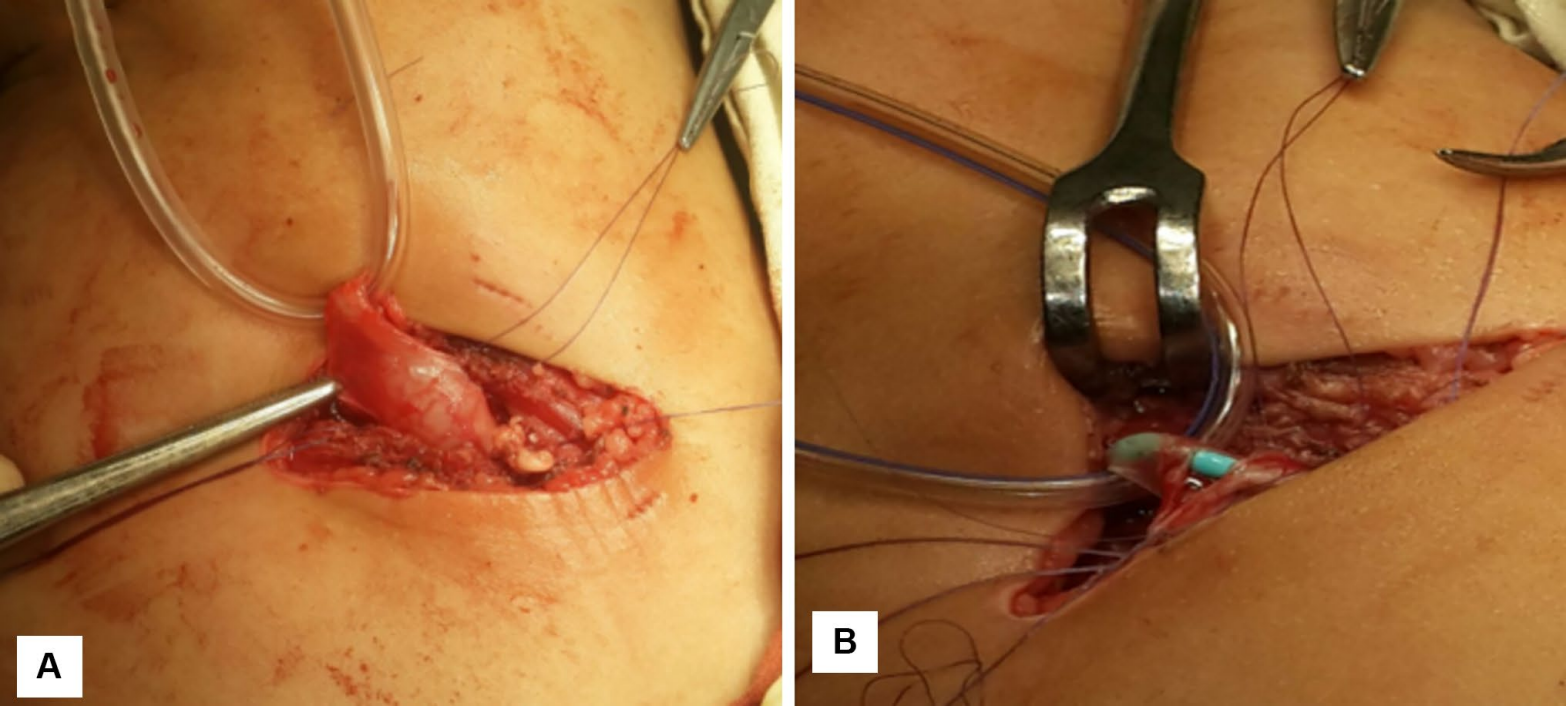
**A** Exposure of UPJ and dissection of the renal pelvis. **B** The anastomosis was completed with inserting a ureteric stent

The flank skin incision was made at the tip of the 12th rib. The external oblique, latissimus dorsi, internal oblique, and serratus posterior inferior muscles were dissected. The transversalis muscle was thin and could be divided using digital dissection. The peritoneum was identified and retracted medially. Gerota’s fascia was then encountered and opened longitudinally to gain access to the perinephric space. Exposure to the UPJ was achieved. The ureter was dissected cephalically towards the renal pelvis (Fig. 2A), while preserving a significant amount of periureteral tissue to ensure an adequate blood supply to the ureter. The UPJ was identified, and the renal pelvis was dissected free from the surrounding peripelvic tissue. The reduction of the renal pelvis was achieved by excising the dilated renal pelvis up to approximately 2 cm from the calyceal infundibula. When the UPJ was excised, the proximal ureter was spatulated on its lateral side. The anastomosis was performed using 5/0 running absorbable sutures to ensure a watertight closure (Fig. 2B). An indwelling ureteral stent was left in place, a tube drain was left in the operative bed and the wound was then closed in layers.

### In both groups

The drains were removed before discharge, and the stent was removed 4–6 weeks post-operatively. Repeat ultrasonography was performed after pyeloplasty at 1 month, 3 months, 6 months, and 1 year. The diuretic DTPA renogram was also repeated 1 year after the operative correction. Descriptive statistics were used to summarize the baseline characteristics, study demographics, clinical data, intraoperative findings, immediate and long-term follow-up.

### Statistics

Statistical analysis was performed using commercially available software, including Excel 2019 and SPSS 22. Continuous variables were expressed as means SD, while categorical variables were expressed as percentages. Comparative analysis of qualitative data was conducted using the Chi-square test. A *p* value < 0.05 was considered significant.

## Results

During the study period, a total of 65 patients with UPJO and renal function ≤ 10% were treated in the pediatric surgery departments, using either laparoscopic assisted dismembered pyeloplasty or open methods. The annual prevalence rate was 3.25 cases per year. Out of the 65 cases, 40 underwent laparoscopic assisted pyeloplasty, and 25 underwent open pyeloplasty. A clear male predominance was observed in both groups. In group A, out of the 40 participants, 32 were males and 8 were females. Similarly, in group B, out of the 25 participants, 18 were males and 7 were females. The left side was more commonly affected in both groups, with 22 out of 40 cases in group A and 12 out of 25 cases in group B. On the other hand, the right side was affected in 12 cases in group A and 10 cases in group B. Bilateral cases were observed in nine cases in both groups. The mean age at the time of intervention was 3 ± 1.2 years in group A and 2 ± 0.92 years in group B (Table 1). In group A, 15 patients were diagnosed with prenatal hydronephrosis, and kidney function was found to be poor after birth. The remaining 25 cases were diagnosed during infancy and childhood. Out of these, 10 cases were discovered accidentally, seven cases were characterized by recurrent fever, six cases presented with abdominal pain, and two cases presented with abdominal swelling. In group B, 8 cases were discovered prenatally, and the remaining 17 cases were discovered during infancy and childhood. Of these, seven cases were accidentally discovered, five cases were identified due to recurrent fever, four cases presented with abdominal pain, and one case presented with abdominal swelling (Table 2).

**Table 1:** Demographic data of cases

Demographic data	Group A Lap assisted pyeloplasty (40)	Group B Open pyeloplasty (25)	*P* value
Age of interference/years	Mean 3 ± 1.2	Mean 2 ± 0.92	0.081*
Sex			
Male	32 (80%)	18 (72%)	0.460^#^
Female	8 (20%)	7 (28%)	
Side			
Right side	12 (30%)	10 (40%)	0.705^#^
Left side	22 (55%)	12 (48%)	
Bilateral	6 (15%)	3 (12%)	

*p* < 0.05 is significant

*Student *T* test

^#^Chi-square test

**Table 2:** Clinical presentations of two groups

Clinical data	Group A (*n* = 40)	Group B (*n* = 25)	*P* value
Prenatal diagnosis	15 (37.5%)	8 (32%)	0.991
Accidently discovered	10 (25%)	7 (28%)	
Recurrent attacks of fever	7 (17.5%)	5 (20%)	
Vague abdominal pain	6 (15%)	4 (16%)	
Abdominal swelling	2 (5%)	1 (4%)	

*p* < 0.05 is significant

No complications or difficulties were encountered during the surgical procedures. The mean operative time was significantly shorter in group A (90 ± 12) minutes, while in group B, it was (120 ± 11) minutes (Table 3). The post-operative hospital stay was significantly shorter in group A (5 ± 1 days) than in group B (7 ± 2 days). However, five cases of minor leakage were detected through the drain, three cases were observed in group A, while two were observed in group B. Fortunately, all cases were successfully managed conservatively. Seroma and wound infection occurred in two cases in group A and four cases in group B. Overall, 62 out of 65 cases (95.3%) showed significant improvement. Unfortunately, during the post-operative follow-up period, two patients (5%) in group A and one patient (4%) in group B experienced kidney function loss and required nephrectomy (Table 4). The nephrectomy procedure was safely and easily performed laparoscopically in group A due to reduced scarring and fibrosis resulting from the primary operation.

**Table 3:** Operative time in the two groups

		Group A (*n* = 40)	Group B (*n* = 25)	*P* value
Operative time/minutes	Mean	90 ± 12	120 ± 11	** < 0.001**

*p* < 0.05 is significant (in bold)

**Table 4:** Post-operative data in the two groups

Post-operative data	Group A (*n* = 40)	Group B (*n* = 25)	*P* value
Post-operative hospital stays (mean/day)	5 ± 1	7 ± 2	0.042
Leakage of the anastomosis	3 (7.5%)	2 (8%)	1.00
Seroma of the wound	2 (5%)	4 (20%)	0.194
Loss of function	2 (5%)	1 (4%)	1.00

*p* < 0.05 is significant

During the follow-up period, which ranged from 12 to 75 months in group A and 12–80 months in group B, all clinical symptoms were improved in all cases, the renal assessment parameters significantly improved in both groups after 12 months. In group A, the mean Anteroposterior Pelvis Diameter (APPD) was 38 ± 7.2 mm and decreased to 16 ± 3.1 mm post-operatively. The mean renal parenchymal thickness was 3.1 ± 0.9 mm and increased to 8.3 ± 1.0 mm post-operatively. The mean split renal function was 7.9 ± 1.3%, which increased to 22.22 ± 6.3%. In group B, the mean APPD was 40 ± 7.2 mm and decreased to 14 ± 2.1 mm post-operatively. The mean renal parenchymal thickness was 3.0 ± 1.2 mm and increased to 8.1 ± 1.23 mm post-operatively. The mean split renal function was 8.1 ± 1.1%, which increased to 24.20 ± 5.1%. However, the differences between the groups in terms of pre-operative and post-operative renal assessment parameters were statistically insignificant (Table 5).

**Table 5:** Comparison between pre-operative and post-operative renal assessments in the two groups

	Group A (40)	Group B (25)	*T* (test value)	*P* value*
APPD				
Pre-op	38 ± 7.2 mm	40 ± 7.2 mm	1.09	0.280
Post-op	16 ± 3.1 mm	14.6 ± 3.2 mm	1.750	0.085
^♦^*P* value	< 0.001	< 0.001		
Renal parenchymal thickness				
Pre-op	3.1 ± 0.9 mm	3.0 ± 1.2 mm	0.383	0.703
Post-op	8.3 ± 1.0 mm	8.1 ± 1.23 mm	0.319	0.951
^♦^*P* value	< 0.001	< 0.001		
DPTA renal scan				
Pre-op	7.9 ± 1.3%	8.1 ± 1.1%	0.639	0.525
Post-op	22.2 ± 6.3%	24.2 ± 5.1%	2.181	0.051
^♦^*P* value	< 0.001	< 0.001		

*p* < 0.05 is significant

*T* test value

*Student *T* test

^♦^Paired *T* test

## Discussion

Ureteropelvic junction obstruction (UPJO) is one of the most common obstructive malformations in pediatric urology [5]. The incidence of this condition ranges from 1 per 500 births [2]. This pathology is most commonly observed in boys (60–75%). According to the literature, the left side is the most frequently affected (60%). It can also be bilateral in 10–20% of cases [8–10]. These data are consistent with our results.

Antenatal diagnosis has been made in over 50% of cases [9, 10]. In our study, antenatal diagnosis was made in 15 out of 40 patients (37.5%) in group A and 8 out of 25 in group B. This percentage difference can be explained by several factors. First, the small sample size of our study may have contributed to the variation. In addition, inadequate prenatal care in developing countries, the operator-dependent nature of ultrasound scans, and low socio-economic level, can all hinder the thorough monitoring of pregnancies.

The management of patients with UPJO has evolved considerably over the past few decades [11, 12]. Laparoscopy is one of the preferred methods for managing many urological diseases. While open surgical procedures are still considered the gold standard, they are being replaced by techniques that offer equivalent success rates, reduced post-operative pain, and shorter hospital stays [13].

Laparoscopic pyeloplasty in children is still controversial due to the challenges of intracorporeal suturing and the limited space within the intraperitoneal cavity. This procedure is difficult to learn and time-consuming [14]. After an initial experience, it was suggested that the laparoscopic approach should not be performed in children younger than 6 months of age, while in our study, laparoscopic procedures have been performed on patients who were more than 3 months. Consequently, the patients who undergo laparoscopic procedures tend to be older than open [15]. Moreover, handling fine suture materials with current laparoscopic instruments remains cumbersome. As originally described by Lee et al. [16], exteriorizing the anastomosis in laparoscopic assisted dismembered pyeloplasty (LADP) overcomes these challenges. The technique is similar to the exteriorization of the loop of the intestine used in gastrointestinal anastomosis [17]. LADP provided less mobilization that has been required to bring the UPJ out of the flank. The duration of the surgery was much shorter than that of a contemporary series of pediatric laparoscopic pyeloplasty. As this procedure does not involve intracorporeal suturing, the learning curve is much shorter compared to that of a complete laparoscopic pyeloplasty [18, 19].

Helal and Daboos [20] utilized laparoscopic assisted pyeloplasty in approximately 20 out of 40 patients. They concluded that this minimally invasive method had a shorter operative time and less post-operative pain. However, the functional results showed no significant difference when compared to the open method. In our study, we adopted the same approach in group A, as the previous researchers and found that this method eliminates the requirement for a large lumbar incision. The mean operative time in the group of LADP was shorter; in our study, it was 90 ± 12 min, while Helal and Daboos [20] series had a longer operative time and it was 120 min. Therefore, we adapted the technique of laparoscopic assisted pyeloplasty in cases of group A because of the benefits of a minimally invasive approach, in addition to easy, safe exploration fewer wound complications, and short hospital stay.

Numerous studies have focused on impaired renal function to establish an algorithm for its evaluation and treatment [2, 7, 21]. However, the management of poorly functioning kidneys, which are associated with less than 10% of UPJO cases, is still debatable [2, 21]. Defining whether these patients would benefit from pyeloplasty and avoid undergoing total nephrectomy. We have reviewed the series in which the authors have defined, in their results, the group of patients considered to have UPJO with impaired renal function. In their series of 142 renal units, Hashim [22] considered renal function to be impaired if it was less than 20%. Bowen [23], Nayyar [24], Nishi [25], Dhillon [26], and Salem [27] also utilized this criterion. UPJO with severely impaired renal function was reported in studies by Lone [28], Ulman [29], and Csaicsich [30]. In their series, the renal function value was found to be less than 15%. Similarly, Abdelaziz [31], Gupta [2], Aziz [7], Bassiouny [32], and Wagner [33] reported a renal function value of less than 10% in their series.

In our series, a cutoff value of 10% was used to define UPJO with impaired function in scintigraphy. Previously therapeutic options included conservative treatment (pyeloplasty), nephrostomy followed by pyeloplasty, and radical treatment (nephrectomy) [28, 32]. However, recent studies with long-term follow-up have shown an improvement in renal function after pyeloplasty in these cases. Thus, nephrectomy is no longer justified [31, 34].

The diameter of the renal pelvis was reduced in all cases in the current study. Specifically, the mean antero-posterior (AP) diameter decreased from 38 ± 7.2 mm and 40 ± 7.2 mm in both groups to 16 ± 3.1 mm and 14 ± 2.1 mm post-operatively. Regarding renal function, our results showed improvement with a mean increase from 7.9 ± 1.3% and 8.1 ± 1.1% pre-operatively to 22.22 ± 6.3% and 24.20 ± 5.1% post-operatively. Other studies have shown an improvement in RF following pyeloplasty. Wagner [33] conducted a study on the outcomes of pyeloplasty in 32 patients who were divided into 3 groups: group I (RF > 40%), group II (10% < RF < 40%), and group III (RF < 10%). A significant improvement in RF was observed in patients who underwent pyeloplasty 12 months after the procedure. Specifically, patients with an initial RF level < 10% experienced a mean post-operative RF of 36%. Abdelaziz [31] conducted a prospective study on 25 patients with UPJO and a split renal function (SRF) of less than 10% who underwent pyeloplasty. The study observed an improvement in FR, with an average increase from 5% pre-operatively to 21% post-operatively after 6 months of surgery, and 20% after 12 months (Table 6).

**Table 6:** The various studies have emphasized the impact of pyeloplasty on a kidney with UPJO and renal function ≤ 10%

	RF	Number	Year	Follow-up mean	Post-op RF
Gupta [2]	≤ 10%	17	2001	27 ± 15 month	31.4 ± 12.8%
Aziz [7]	≤ 10%	12	2002	–	35–40%
Wagner [33]	≤ 10%	4	2008	24 month	41%
Bansal [21]	≤ 10%	6	2012	41.6 month	22.8%
Abdelaziz [31]	≤ 10%	25	2018	–	20%
Our study	≤ 10%	65	2023	27 ± 5 month [12–80]	22.2 ± 6.3% in group A 24.2 ± 0.51% in group B

Although our work provides an answer to the choice of surgical treatment for renal units with UPJO and impaired renal function ≤ 10% of overall function, it is primarily limited by the short follow-up period. This limitation can affect the perception of late complications and the necessity of nephrectomy after a prolonged period of progression.

## Conclusion

Laparoscopic assisted pyeloplasty is an effective treatment for patients with poorly functioning kidneys, especially those with less than 10% function. While this surgical procedure requires shorter operative times, it yields functional outcomes that are comparable to open approach. Our work can be considered a blueprint for future multicenter, prospective studies with a larger sample size and prolonged follow-up period.

## Data Availability

The datasets used and/or analyzed during the current study are available from the corresponding author. However, due to the medicolegal aspect of the hospital policy, they cannot be sent.
